# Draft Genome Sequence of Stenotrophomonas pavanii Strain MHSD12, a Bacterial Endophyte Associated with Dicoma anomala

**DOI:** 10.1128/MRA.00550-20

**Published:** 2020-07-09

**Authors:** Mehabo P. Maela, Mahloro H. Serepa-Dlamini

**Affiliations:** a Department of Biotechnology and Food Technology, University of Johannesburg, Johannesburg, South Africa; Portland State University

## Abstract

Stenotrophomonas pavanii strain MHSD12 is an endophyte isolated from Dicoma anomala surface-sterilized leaves. Here, we report its draft genome sequence (4.3 Mb) assembled to 30 contigs, with 3,923 protein-coding genes. The genome sequence highlights important genes for an endophytic lifestyle.

## ANNOUNCEMENT

*Stenotrophomonas* spp. are widespread and were previously isolated from diverse environments, such as soil, sewage, compost, human clinical samples, petrochemical waste, and plants (1). Several *Stenotrophomonas* species have symbiotic interactions with plants and have been isolated as endophytes (2–5). Stenotrophomonas pavanii strain MHSD12 was isolated as an endophyte from healthy surface-sterilized leaves of Dicoma anomala*. Dicoma anomala* aerial tissues were collected from Eisleben, Limpopo Province, South Africa. Leaf samples were serially sterilized with 70% ethanol and 5% sodium hypochlorite (5) and rinsed 3 times with autoclaved tap water. The surface-sterilized leaves were crushed using a sterile mortar and pestle and macerated with phosphate-buffered saline (8 g NaCl, 0.2 g KCl, 1.44 g Na_2_HPO_4_, and KH_2_PO_4_ at pH 7.4). The homogenate was streaked onto nutrient agar and incubated at 28°C for 2 to 7 days, followed by subculturing single colonies 3 times. Stenotrophomonas pavanii strain MHSD12 was initially identified by phylogenetic analysis of its 16S rRNA gene (GenBank accession number MN078164). The genome sequence of *S. pavanii* strain MHSD12 will augment studies on the plant-bacterium interaction and highlight important genes responsible for an endophytic lifestyle.

For genomic DNA extraction, MHSD12 was grown on nutrient broth at 28°C for 48 h. Genomic DNA was extracted using the NucleoSpin microbial DNA extraction kit (Macherey-Nagel, Germany), following the manufacturer’s protocol. The obtained DNA was sequenced on an Illumina platform at a commercial service provider (Agricultural Research Council [ARC], Onderstepoort, South Africa). Paired-end libraries (2 × 150 bp) were generated using the Nextera DNA sample preparation kit (Illumina, USA), and sequencing was performed using the HiSeq 2500 platform.

All of the preannotation analyses were performed on the Galaxy Web server (https://usegalaxy.org) (6), using default parameters. Quality control of the raw reads was performed by FastQC version 0.72 (7). Sequence reads were *de novo* assembled using Unicycler version 0.4.6.0 (8), and the assembly quality was assessed with QUAST version 0.4.6.3 (9). The draft genome sequence of strain MHSD12 was submitted to NCBI for automated annotation using Prokaryotic Genome Annotation Pipeline (PGAP) (10) and the Rapid Annotations using Subsystems Technology (RAST) server (11–13). The genome sequence data of *S. pavanii* strain MHSD12 was submitted to the Type (Strain) Genome Server (https://tygs.dsmz.de) for a whole-genome-based taxonomic analysis with other validly published type strains (14). Additionally, the average nucleotide identity (ANI) value with closely related species was determined using the Orthologous Average Nucleotide Identity Tool (OAT) software (15).

The sequencing platform produced 2,587,651 sequence reads and allowed a 57-fold coverage. The genome sequence was assembled to 30 contigs with an *N*_50_ value of 403,304 bp, a total genome size of 4,385,734 bp, and a G+C content of 67.34%. A total of 4,025 genes, including 3,923 protein-coding genes and 73 RNA genes, were identified. The RAST annotation identified 1,495 subsystems with amino acids and derivatives as the dominant (17%) category, and categories that play a part in plant promotion and growth, such as nitrogen and phosphate metabolism, iron acquisition and virulence, and disease and defense, were identified. The highest similarity was with Stenotrophomonas pavanii strain DSM 25135 which is an endophyte isolated from sugar cane (16); MHSD12 had a 93.2% digital DNA-DNA hybridization value and a 98% ANI value with DSM 25135.

### Data availability.

This whole-genome shotgun project and associated data have been deposited at DDBJ/ENA/GenBank under the accession number JAAKGL000000000, BioProject accession number PRJNA607646, and BioSample accession number SAMN14142486. The version described in this paper is the first version, JAAKGL010000000. The raw sequence reads are available at SRR11955405.
